# Quantifying myocardial fibrosis in hypertensive left ventricular hypertrophy using T1 mapping

**DOI:** 10.1186/1532-429X-14-S1-P172

**Published:** 2012-02-01

**Authors:** Rajesh Janardhanan, Nebiyu Adenaw, Ronny Jiji, Jeremy Brooks, Frederick H Epstein, Christopher M Kramer, Michael Salerno

**Affiliations:** 1 University of Virginia Health System, Charlottesville, VA, USA

## Summary

Diffuse myocardial fibrosis in hypertensive left ventricular hypertrophy (LVH) is not readily detected by conventional late gadolinium enhanced CMR. Our study was aimed to detect diffuse myocardial fibrosis in these patients as compared to age matched normal controls, using a T1 mapping technique. Fibrosis was quantified by calculating the partition coefficient (λ) and volume of distribution (Vd) of gadolinium (Gd) following a bolus injection of Gd. We observed that the values for λ and Vd were higher in LVH than controls. A positive association was also noted between LV mass and λ.

## Background

Diffuse myocardial fibrosis can occur in hypertensive left ventricular hypertrophy (LVH) and is not readily detected by conventional late gadolinium enhanced CMR. We previously described a shortened Modified Look-Locker Inversion Recovery (3-5 MOLLI) T1-mapping technique which can quantify fibrosis by calculating the partition coefficient (λ) and volume of distribution (Vd) of gadolinium (Gd) following a bolus injection of Gd.[1] We hypothesized that this technique could detect fibrosis in subjects with hypertensive LVH and normal ejection fraction. This could have important implications for assessment of diastolic heart failure and to monitor benefits of anti-fibrotic, anti-hypertensive therapy. We aimed to detect diffuse myocardial fibrosis in hypertensive patients with LVH as compared to age matched normal controls.

## Methods

T1 mapping was performed in 11 subjects with hypertensive LVH (53±16 years) and normal ejection fraction, and 7 age-matched healthy volunteers (50±10 years) on a Siemens 1.5T Avanto using 3-5 MOLLI (11 heart beats, 2 inversions, 3 recovery beats, 8 images). Patients with known coronary disease, significant valvular disease, and other causes of LVH were excluded. LV mass and function was assessed by SSFP cine imaging. MOLLI sequence parameters included: TE/TR/FA 1.1 ms/2.5ms/35°, FOV= 340 x 260, resolution 1.8mm x 1.8mm, thickness 8mm. T1 was determined pre-contrast and 10,15 and 20 minutes following injection of 0.15 mmol/kg Gd-DTPA. Hematocrit (Hct) was measured in all subjects. T1 maps were calculated and manually segmented using an in-house MATLAB program. λ was determined from the slope of a plot of 1/T1 of the myocardium versus 1/T1 of the blood. Vd was calculated as (1-Hct)*λ. Values were compared between groups using 2-tailed unpaired t-tests.

## Results

The LVH group had significantly higher blood pressure and LV mass than age-matched controls (Table). Heart rate, Hct and creatinine were similar between groups. Values for λ and Vd were higher in LVH (0.49±0.04 and 0.31±0.02) than controls (0.44±0.01 and 0.27±0.01) (p=0.003 and 0.004) respectively (Figure). There was a positive association between LV mass and λ (Spearman rho=0.66; p=0.01).

**Table 1:** Baseline characteristics of hypertensive LVH subjects and controls

	Hypertensive LVH (n=11)	Age matched controls (n=7)	P value
Sex	7 females; 4 males	6 females; 1 male	
Age (yrs)	53±16	50±10	0.63
Systolic BP (mm Hg)	151±15	120±20	0.002
Diastolic BP (mm Hg)	82±13	62±9	0.002
LV Mass (g)	165±46	91±21	0.001
Ejection fraction (%)	64±7	61±5	0.33
Heart Rate (beats/min)	75±15	68±9	0.28
Hematocrit (%)	37.8±4.0	38.0±2.9	0.94
Creatinine (mg/dL)	0.96±0.28	0.73±0.08	0.06
eGFR	79±16	72±15	0.50

**Figure 1:**
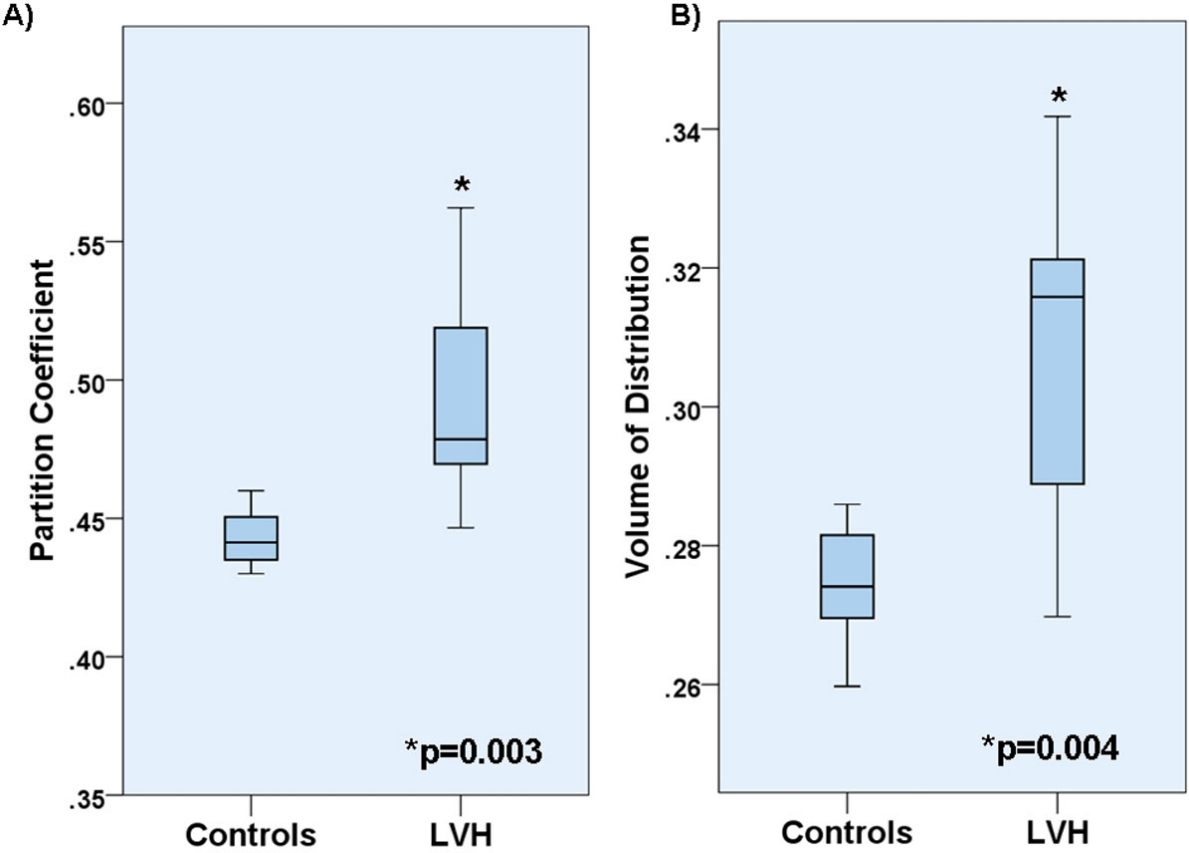
Partition coefficient and volume of distribution of Gadolinium for subjects with hypertensive LVH versus healthy age matched controls

## Conclusions

Determination of λ and Vd by T1 mapping after Gd bolus with a reduced breath-hold 3-5 MOLLI pulse sequence is a robust method capable of quantifying diffuse myocardial fibrosis in hypertensive patients with LVH and normal ejection fraction. λ correlates with LV mass. These findings support the application of T1 mapping to monitor therapies that regress hypertrophy and reduce fibrosis in hypertensive heart disease.

## Funding

Internal departmental funding
